# Multifunctional Gold-Mesoporous Silica Nanocomposites for Enhanced Two-Photon Imaging and Therapy of Cancer Cells

**DOI:** 10.3389/fmolb.2016.00001

**Published:** 2016-02-03

**Authors:** Jonas G. Croissant, Christian Qi, Marie Maynadier, Xavier Cattoën, Michel Wong Chi Man, Laurence Raehm, Olivier Mongin, Mireille Blanchard-Desce, Marcel Garcia, Magali Gary-Bobo, Jean-Olivier Durand

**Affiliations:** ^1^Institut Charles Gerhardt Montpellier, UMR-5253 CNRS-UM2-ENSCM-UM1Montpellier, France; ^2^NanoMedSyn, 2 - Faculté de PharmacieMontpellier, France; ^3^Institut NEEL, CNRS, Université Grenoble AlpesGrenoble, France; ^4^Institut Des Sciences Chimiques de Rennes, CNRS UMR 6226 Université Rennes 1Rennes, France; ^5^University of Bordeaux, Institut des Sciences Moléculaires, UMR CNRS 5255Talence, France; ^6^Institut des Biomolécules Max Mousseron UMR 5247 CNRS; UM 1; UM 2 - Faculté de Pharmacie, Université MontpellierMontpellier, France

**Keywords:** two-photon, gold nanoparticles, organic-inorganic, photodynamic therapy, mesoporous silica nanoparticles

## Abstract

Three dimensional sub-micron resolution has made two-photon nanomedicine a very promising medical tool for cancer treatment since current techniques cause significant side effects for lack of spatial selectivity. Two-photon-excited (TPE) photodynamic therapy (PDT) has been achieved via mesoporous nanoscaffolds, but the efficiency of the treatment could still be improved. Herein, we demonstrate the enhancement of the treatment efficiency via gold-mesoporous organosilica nanocomposites for TPE-PDT in cancer cells when compared to mesoporous organosilica particles. We performed the first comparative study of the influence of the shape and spatial position of gold nanoparticles (AuNPs) with mesoporous silica nanoparticles (MSN) functionalized with thiol groups and doped with a two-photon electron donor (2PS). The resulting multifunctional nanocarriers displayed TPE-fluorescence and were imaged inside cells. Furthermore, mesoporous organosilica NPs decorated gold nanospheres (AuNSs) induced 63 percent of selective killing on MCF-7 breast cancer cells. This study thus provides insights for the design of more effective multifunctional two-photon-sensitive nanocomposites via AuNPs for biomedical applications.

## Introduction

Thanks to its unique spatio-temporal accuracy two-photon nanomedicine is emerging as a very promising medical tool for cancer treatment as witnessed by the recently reported nanodevices with two-photon excitation (TPE) (Lin et al., 2010; Cheng et al., 2011; Gary-Bobo et al., 2011; Chen et al., 2012, 2014; Li et al., 2012; Zhao et al., 2012; Croissant et al., 2013, 2014a,b, 2015; Jiang et al., 2013). Intrinsic properties of this non-linear optical technology provide a three-dimensional resolution of the irradiation, while near-infrared (NIR) TPE enables a deeper tissue penetration (down to 2 cm), lower scattering, and safer treatment than UV-visible excitation (Collins et al., 2008; Starkey et al., 2008). This unparalleled spatial and temporal selectivity of TPE-nanomedicine thus appears to be an ideal alternative to current cancer therapies (e.g., chemotherapy) which lack the necessary spatial selectivity to lower or avoid side-effects on healthy tissues and organs. In this context, pathologies such as retinoblastoma, skin, and breast cancers, to name a few, could be treated much more precisely via two-photon technology and smart nanodevices.

Recently, mesoporous silica nanoparticles (MSN) have been applied *in-vitro* for TPE-fluorescence imaging and photodynamic therapy (PDT) (Qian et al., 2012; Mauriello-Jimenez et al., 2015; Vaillant et al., 2015) two-photon-triggered drug release (Guardado-Alvarez et al., 2013, 2014) and drug delivery (Croissant et al., 2013, 2014a). Mesoporous silica and organosilica nanomaterials are indeed excellent candidates for efficient nanomedicine since they are biocompatible (Tang et al., 2012; Chen et al., 2013a; Knezevic and Lin, 2013), endocytosed and exocytosed by cells (Yanes et al., 2013), excreted (Lu et al., 2010; He et al., 2011), and suitable platforms for multifunctional theranostics (Ambrogio et al., 2011; Rosenholm et al., 2011; Chen et al., 2013b; Mai and Meng, 2013; Mamaeva et al., 2013; Argyo et al., 2014). Nonetheless, the efficiency of the 2PE treatment often needs to be improved because of low two-photon absorption cross-sections. One option consists of loading a significant amount of photosensitizer in MSN in order to increase the overall cross-section in a single NP, but the loading capacity is generally limited to few weight percents (3–8 wt%). Besides, the loaded photosensitive molecules often leak out from the nanocarrier which in turns decreases the intended biological efficacy. To circumvent this problem, photosensitive molecules could be covalently attached to the mesoporous silica framework, but it is generally challenging to obtain high wt% of photosensitizer while retaining high porosities.

In order to further improve the TPE-PDT, we recently reported the use of AuNPs with two-photon-sensitive non-porous bridged silsesquioxanes NPs which possessed 80% of 2PS in the structure (Croissant et al., 2015). Since one-photon photothermal and photodynamic therapies have been reported *in-vitro* and *in-vivo* via gold nanorods (AuNRs) encapsulated in MSN (Zhang et al., 2012; Monem et al., 2014), we expected that specific designs of two-photon-responsive gold-MSN nanocomposites could lead to higher anticancer efficiencies.

Herein we report enhanced efficiencies of two-photon nanomedicine via multifunctional gold-mesoporous organosilica nanocomposites for TPE-PDT in cancer cells. A library of two-photon-sensitive mesoporous organosilica (M2PS), gold-M2PS NPs is described and systematically applied for TPE-fluorescence and therapy on MCF-7 breast cancer cells. The two-photon photosensitizer (2PS) was covalently linked with high contents (8–20 wt%) within the walls of ordered mesopores. This study demonstrates cellular uptakes of the nanocomposites via TPE-fluorescence, and TPE-PDT produced up to 71% of spatiotemporal cell death.

## Materials and methods

### Materials

Tetraethoxysilane (TEOS), mercaptopropyltrimethoxysilane (MPTMS), cetyl-trimethylammonium bromide (CTAB), sodium hydroxide, ammonium nitrate, potassium tetrachloroaurate, sodium citrate trihydrate, ascorbic acid, and sodium borohydride were purchased from Sigma-Aldrich. Absolute ethanol was purchased from Fisher Chemicals.

Dynamic light scattering analyses were performed using a Cordouan Technologies DL 135 Particle size analyzer instrument. TEM analysis were performed on a JEOL 1200 EXII instrument.

### Synthesis of M2PS NPs

A mixture of CTAB (250 mg, 6.86^*^10^−1^ mmol), distilled water (120 mL), and sodium hydroxide (875 μL, 2 M) was stirred at 80°C during 50 min at 700 rpm in a 250 mL three neck round bottom flask. Then, TEOS (1 mL) along with an alcoholic solution of the 2PS precursor (177 mg, 1.29^*^10^−1^ mol, in 500 μL of EtOH) (Croissant et al., 2015) were added to the aforementioned solution, and the condensation process was conducted for 2 h. Then, the solution was cooled to room temperature while stirring; fractions were gathered in polypropylene tubes, and NPs were collected by centrifugation during 15 min at 21 krpm. The sample was extracted twice with an alcoholic solution of ammonium nitrate (6 g.L^−1^), and washed three times with ethanol, water, and ethanol. Each extraction involved a sonication step of 30 min at 50°C to remove the CTAB surfactant; the collection was carried out in the same manner. The as-prepared material was dried for few hours under vacuum.

### Synthesis of Au@M2PS NPs

A mixture of water (25 mL), ethanol (10 mL), and CTAB (160 mg, 4.40^*^10^−1^ mmol) was prepared in a three neck 50 mL round bottom flask, and stirred at 70°C. Then an aqueous solution of potassium tetrachloroaurate (13 mg, 3.45^*^10^−2^ mmol in 1 mL) was injected, and sodium hydroxide (100 μL, 2 M) was added to induce the instantaneous nucleation of nanoparticles (Croissant and Zink, 2012). After 30 s, hydrochloric acid (18 μL, 2 M) was added for a controlled sol-gel process. The nanoparticles growth was conducted for 20 min under a 600 rpm stirring speed, and the temperature was then set at 80°C. Then, TEOS (450 μL, 2.01 mmol) and the 2PS precursor (89 mg, 6.45^*^10^−2^ mmol, in 900 μL of anhydrous ethanol) were added dropwise to the aforementioned stirring solution, followed by sodium hydroxide (100 μL, 2 M) to grow the porous M2PS shell onto gold nanocrystals. The condensation process was conducted for 1 h. Then, the solution was cooled to room temperature while stirring; fractions were gathered in polypropylene tubes, and NPs were collected by centrifugation during 15 min at 21 krpm. Extractions and following steps were identical to those of M2PS NPs.

### Synthesis of MSNSH NPs

A mixture of CTAB (250 mg, 6.86^*^10^−1^ mmol), distilled water (120 mL), and sodium hydroxide (875 μL, 2 M) was stirred at 80°C during 50 min at 700 rpm in a 250 mL three neck round bottom flask. Then, TEOS (1 mL) was added to the aforementioned solution, and after 6 min, mercaptopropyltriethoxysilane was added (100 μL, 5.38^*^10^−1^ mmol), and the condensation process was conducted for 2 h. Then, the solution was cooled to room temperature while stirring; fractions were gathered in polypropylene tubes, and NPs were collected by centrifugation during 15 min at 21 krpm. Extractions and following steps were identical to those of M2PS NPs.

### Synthesis of M2PSSH NPs

A mixture of CTAB (250 mg, 6.86^*^10^−1^ mmol), distilled water (120 mL), and sodium hydroxide (875 μL, 2 M) was stirred at 80°C during 50 min at 700 rpm in a 250 mL three neck round bottom flask. Then, TEOS (1 mL) along with an alcoholic solution of the 2PS precursor (177 mg, 1.29^*^10^−1^ mol, in 500 μL of EtOH) were added to the aforementioned solution. After 6 min, mercaptopropyl-triethoxysilane was added (100 μL, 5.38^*^10^−1^ mmol), and the condensation process was conducted for 2 h. Then, the solution was cooled to room temperature while stirring; fractions were gathered in polypolypropylene tubes, and NPs were collected by centrifugation during 15 min at 21 krpm. Extractions and following steps were identical to those of M2PS NPs.

### Synthesis of Au nanospheres

The elaboration of gold nanospheres (AuNSs) was performed via a modified Turkevich method (Kimling et al., 2006). A mixture of water (100 mL), and potassium tetrachloroaurate (55 mg, 1.38^*^10^−1^ mmol) was refluxed and stirred in a three neck 250 mL round bottom flask. Then, an aqueous solution of sodium citrate (155 mg, 5.3^*^10^−1^ mmol in 3 mL) was quickly injected through two syringes (2^*^1.5 mL). The reaction was conducted for 10 min, and the solution was cooled to room temperature. The resulting solution of AuNSs was used without further modification.

### Synthesis of MSNSH@Au NPs

A mixture of MSNSH NPs (25 mg) and deionized water (5 mL) was sonicated 5 min, then a fraction of the AuNSs solution (20 mL) was added. The solution was stirred 20 min at 60°C, and cooled down 40 min to room temperature before being centrifuged 15 min at 21 krpm. Supernatants were removed, the compound was washed twice with ethanol, and was collected via centrifugation for 10 min at 21 krpm. The as-prepared material was dried for few hours under vacuum.

### Synthesis of M2PSSH@Au NPs

A mixture of M2PSSH NPs (25 mg) and deionized water (5 mL) was sonicated 5 min, then a fraction of the AuNSs solution (20 mL) was added. The solution was stirred 20 min at 60°C, and cooled down 40 min to room temperature before being centrifuged for 15 min at 21 krpm. Supernatants were removed, the compound was washed twice with ethanol, and was collected via centrifugation for 10 min at 21 krpm. The as-prepared material was dried for few hours under vacuum.

### Synthesis of Au nanorods

The elaboration of gold nanorods (AuNRs) was performed via a seed-growth method reported by Gorelikov and Matsuura [44]. First, a mixture of CTAB (182 mg, 5.0^*^10−1 mmol), distilled water (2.5 mL), and potassium tetrachloroaurate (1.5 mL, 0.001 M) was stirred (1 cm stir bar at 1400 rpm) in a 5 mL round bottom flask. Then, an ice-cooled aqueous solution of sodium borohydride (600 μL, 0.01 M) was injected, and the reaction was allowed to stir for 2 min before lowering the stirring speed to 500 rpm. The seed solution was used in the following hour without further purification. Separately, a mixture of cetyltrimethylammonium bromide [911 mg, CTAB (SIGMA reference 6269), 2.5 mmol], distilled water (24 mL), silver nitrate (700 μL, 4 mM), and potassium tetrachloroaurate (1.250 mL, 15 mM) was stirred (2.5/0.7 cm stir bar at 1000 rpm) in a 50 mL round bottom flask at 30°C. Second, ascorbic acid was added dropwise (313 μL, 0.08 M) to reduce Au(III) ions in Au(I) species, and gold seeds (250 μL) were added in the mixture to trigger the anisotropic growth on seeds. The solution turned red after ca. 15 min and the reaction was conducted for a total of 40 min.

### Synthesis of MSNSH@AuNRs NPs

A mixture of MSNSH NPs (25 mg) and deionized water (5 mL) was sonicated for 5 min, then a fraction of the AuNRs solution (20 mL) was added, followed with sodium hydroxide (1 mL, 0.2 M). The solution was stirred for 20 min at 60°C, neutralized with hydrochloric acid addition (0.2 M), and cooled down for 40 min to room temperature before centrifugation for 15 min at 21 krpm. Supernatant were removed, the compound was washed twice in ethanol, and once with acetone, and was collected via centrifugation for 10 min at 21 krpm. The as-prepared material was dried for few hours under vacuum.

### Synthesis of M2PSSH@AuNRs NPs

A mixture of M2PSSH NPs (25 mg) and deionized water (5 mL) was sonicated for 5 min, then a fraction of the AuNRs solution (20 mL) was added, followed with sodium hydroxide (1 mL, 0.2 M). The solution was stirred for 20 min at 60°C, neutralized with hydrochloric acid addition (0.2 M), and cooled to room temperature for 40 min before centrifugation for 15 min at 21 krpm. Super-natants were removed, the compound was washed twice in ethanol, once with acetone, and was collected via centrifugation for 10 min at 21 krpm. The as-prepared NPs were dried under air flow for few hours.

### TPE photodynamic therapy of cancer cells

Human breast cancer cells MCF-7 (purchased from ATCC) were cultured in DMEM added with 10% fetal bovine serum and 50 μg.mL^−1^ gentamycin. Cells were allowed to grow in humidified atmosphere at 37°C under 5% CO2. For TPE photodynamic therapy of cancer cells, MCF-7 cells were seeded into a 384 multiwell glass-bottomed plate (thickness 0.17 mm), with a black polystyrene frame, 2000 cells per well in 50 μL of culture medium, and allowed to grow for 24 h. Then, cells were incubated 20 h with NPs at the final concentration of 40 μg.mL^−1^ and submitted or not to TPE performed on a confocal microscope Carl Zeiss Microscope (laser power input 3W). Half of the well was irradiated at 760 nm by three scans of 1.57 s duration in 4 different areas of the well. The laser beam was focused by a microscope objective lens (Carl Zeiss 10 × /0.3 EC Plan-Neofluar). The scan size does not allow irradiating more areas without overlapping. After 2 days, the MTS assay was performed and was corrected according to the following formula Abs _no laser_ − 2 × (Abs _no laser_- Abs _laser_).

### TPE imaging of cancer cells

The day prior to the experiment, MCF7 cells were seeded onto bottom glass dishes (World Precision Instrument, Stevenage, UK) at a density of 10^6^ cells.cm^−2^. Adherent cells were then washed once and incubated in 1 mL medium containing NPs at a concentration of 40 μg.mL^−1^ for 20 h. Fifteen min before the end of incubation, cells were loaded with Cell Mask (Invitrogen, Cergy Pontoise, France) for membrane staining at a final concentration of 5 μg.mL^−1^. Before visualization, cells were washed gently with phenol red-free DMEM. Cells were then scanned with a LSM 780 LIVE confocal microscope (Carl Zeiss, Le Pecq, France), at 760 nm with a slice depth (Z stack) of 0.62 μm.

## Results and discussion

**Scheme 1:**
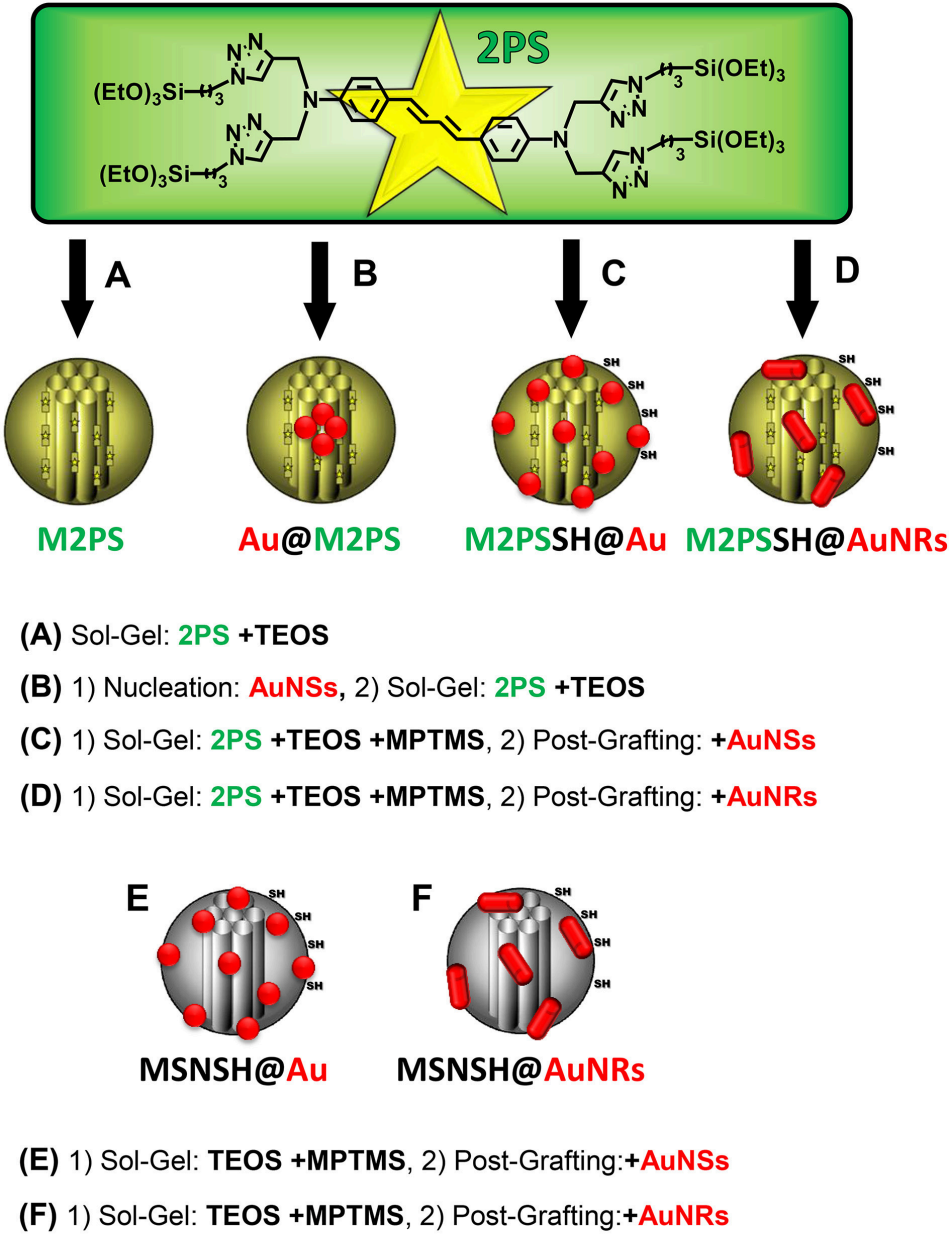
**Representation of the 2PS precursor and of the designed library of gold-mesoporous silica and organosilica nanocomposites for the systematic study of TPE-PDT**. TEOS, tetraethoxysilane; MPTMS, mercaptopropyltrimethoxysilane. **(A)** Sol-Gel: 2PS +TEOS. **(B)** (1) Nucleation: AuNSs, (2) Sol-Gel: 2PS +TEOS. **(C)** (1) Sol-Gel: 2PS +TEOS +MPTMS, (2) Post-Grafting: +AuNSs. **(D)** (1) Sol-Gel: 2PS +TEOS +MPTMS, (2) Post-Grafting: +AuNRs. **(E)** (1) Sol-Gel: TEOS +MPTMS, (2) Post-Grafting:+AuNSs. **(F)** (1) Sol-Gel: TEOS +MPTMS, (2) Post-Grafting:+AuNRs.

A library of six gold-mesoporous silica and organosilica nanocomposites doped with the 2PS (see Scheme 1) will be discussed in regards to the influence of AuNPs on TPE-nanomedicine.

**Figure 1:**
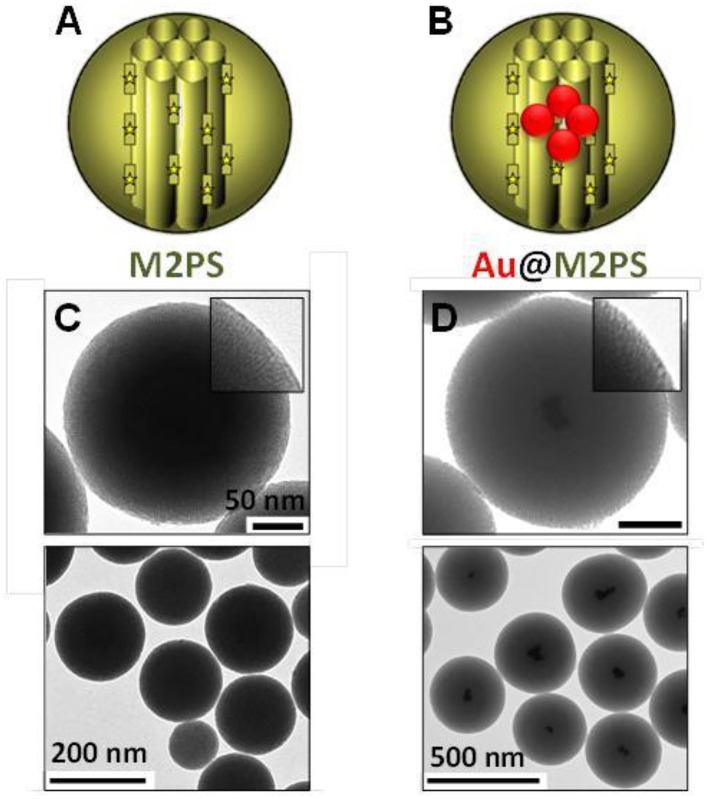
**Schematic representation (A,B) and TEM micrographs of M2PS (C), and Au@M2PS NPs (D)**. Yellow stars represent 2PS fragments.

First, the influence of AuNSs on TPE-imaging and PDT of M2PS was assessed. Two types of nanocarriers were designed via sol-gel process and compared, M2PS and gold core M2PS shell (Au@M2PS) NPs (Figures 1A,B). The M2PS nanomaterial was elaborated by co-condensation of TEOS and the 2PS obtained by click chemistry (Burglova et al., 2014), at 80°C for 2 h in a CTAB-template water/ethanol mixture (5:2, v:v) and sodium hydroxide catalyst. Porous NPs were extracted by sonication in ethanolic ammonium nitrate. Au@M2PS NPs were constructed via a modified one-pot reaction based on the *in-situ* production of AuNSs and subsequent growth of mesoporous organosilica shells (Croissant and Zink, 2012).

**Figure 2:**
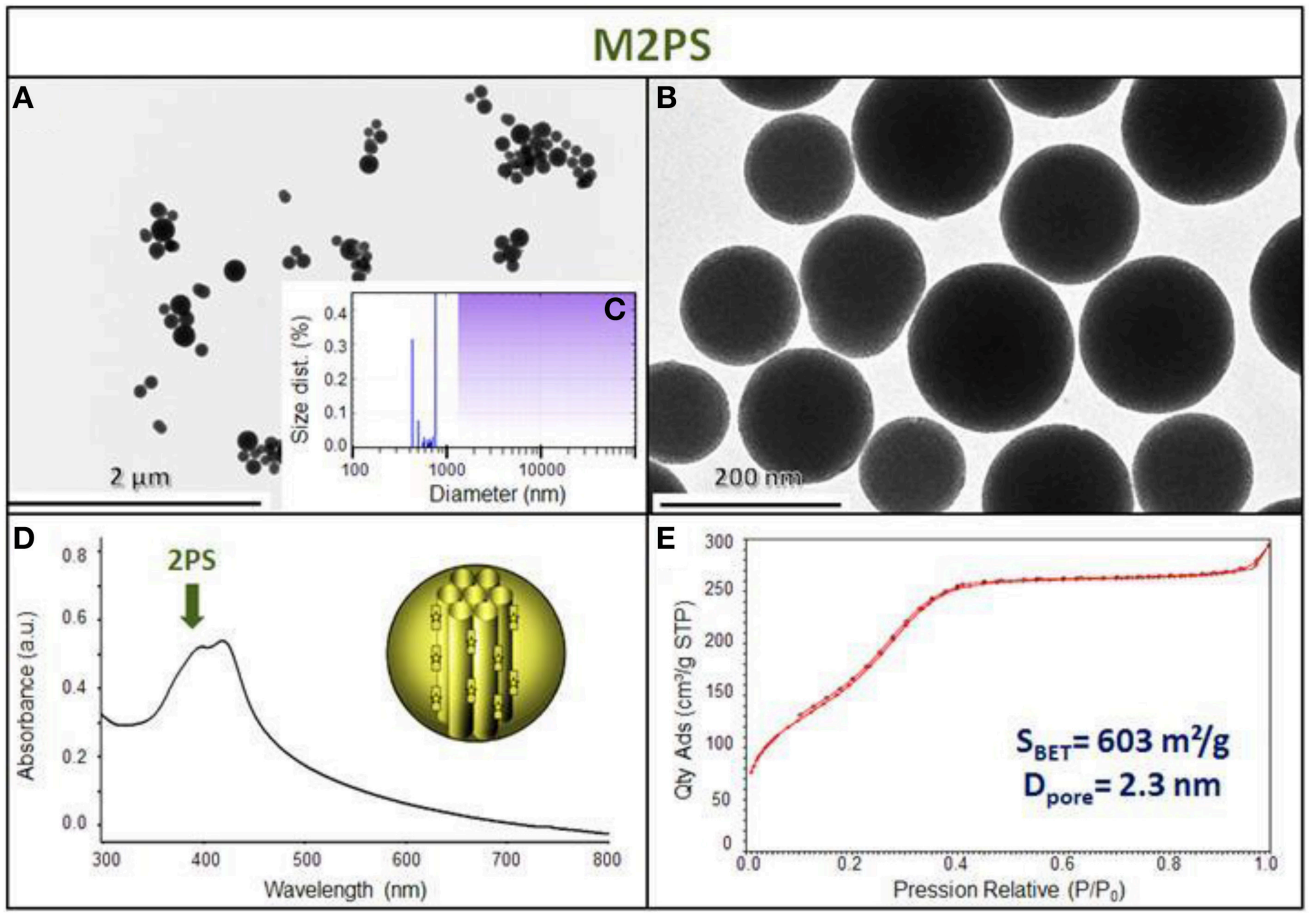
**M2PS NPs characterization via TEM images (A,B), DLS size distribution (C), uv-visible spectroscopy (D), and N_2_-adsorption-desorption (E)**.

**Figure 3:**
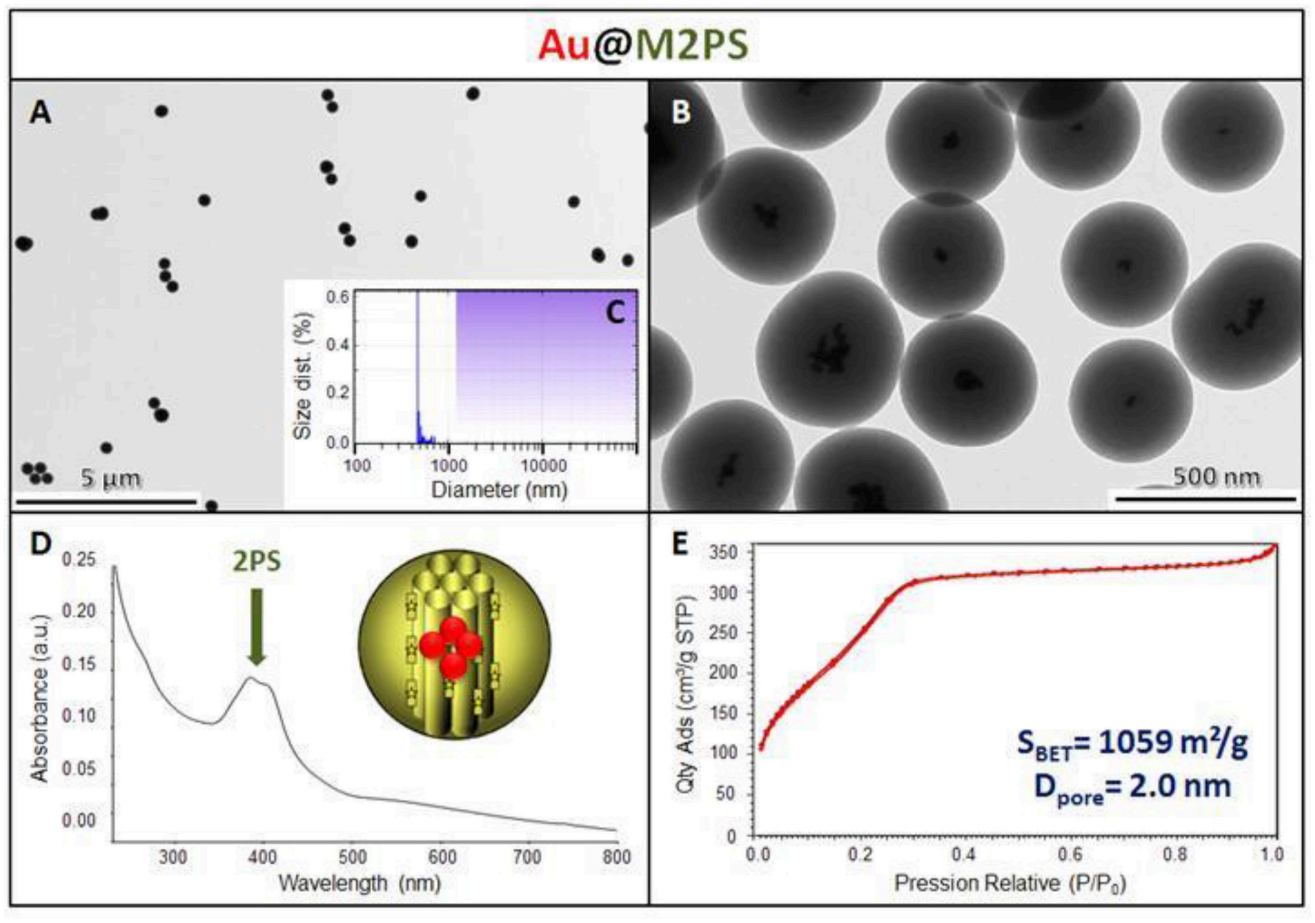
**Au@M2PS NPs characterization via TEM images (A,B), DLS size distribution (C), uv-visible spectroscopy (D), and N_2_-adsorption-desorption (E)**.

The M2PS and Au@M2PS nanocarriers were then characterized via transmission electron microscopy (TEM, Figures 1C,D, 2A,B, 3A,B), which depicted 200 nm monodisperse spherical particles. The narrow size distributions of both systems were confirmed by dynamic light scattering measurement (DLS, Figures 2C, 3C). Moreover, the successful encapsulation of the 2PS moieties was demonstrated by the absorption band of the 2PS (λ_max_ = 385 nm) on the UV-visible spectra of the nanomaterials (Figures 2D, 3D). The 2PS content in M2PS and Au@M2PS NPs were determined to be of 17 and 20 wt% via elemental analysis of nitrogen (14 atoms per 2PS molecule, Table S1). Several gold cores were readily visible on TEM micrographs of Au@M2PS and the typical plasmonic band was observed from 500 to 600 nm on the uv-visible spectrum (Figure 3D). Nitrogen-sorption analysis calculated surface areas of 603 and 808 m^2^.g^−1^ with the Brunauer-Emmett-Teller (BET) theory for M2PS and Au@M2PS, respectively, with 2.0 to 2.3 nm pore diameters according to the Barret-Joyner-Halenda (BJH) method (Figures 2E, 3E). A gold core content of 4.7 wt% via energy dispersive spectroscopy (EDS, Table S2).

**Figure 4:**
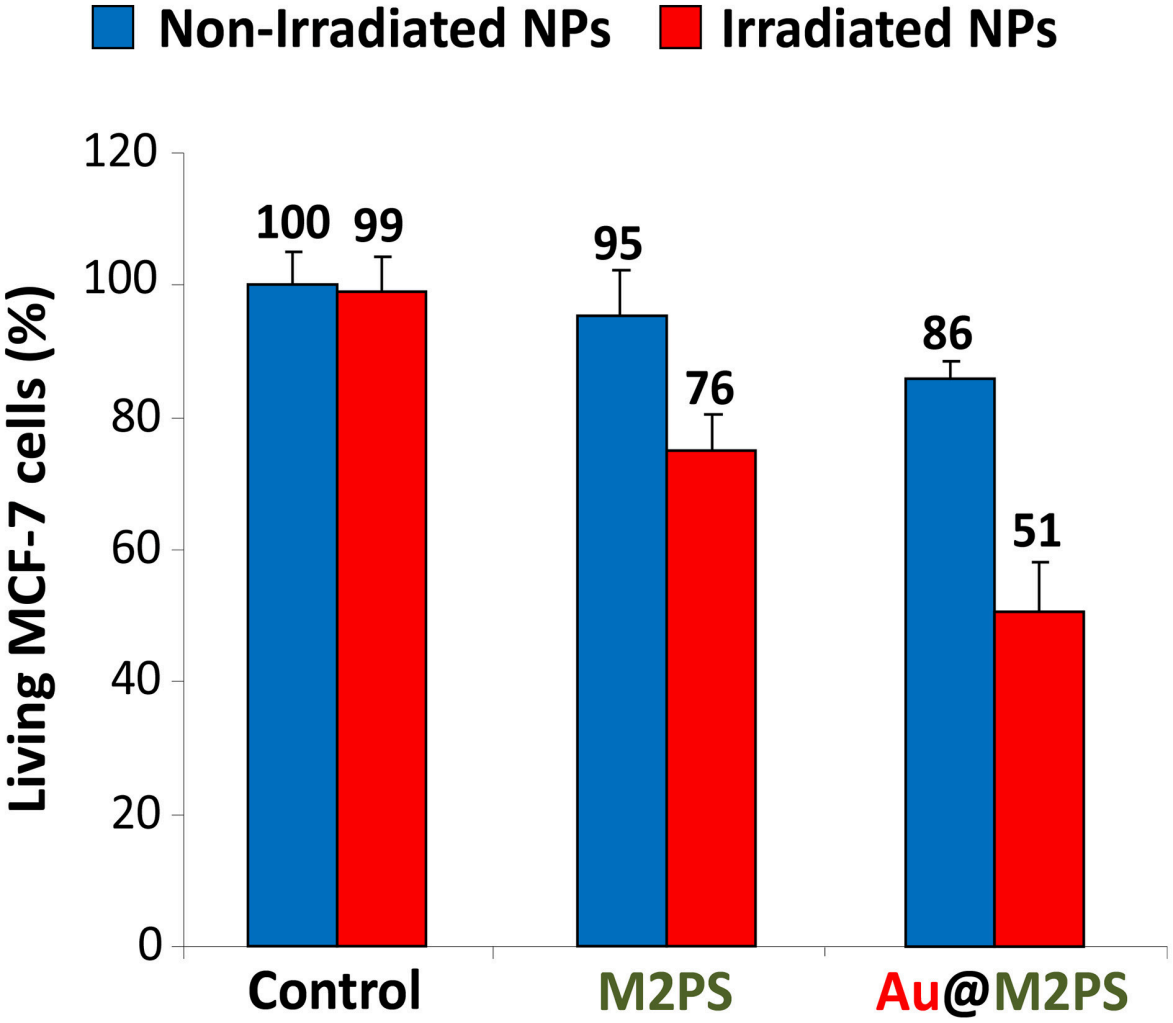
**TPE-PDT via M2PS and Au@M2PS NPs (40 μg.mL^−1^) in MCF-7 cells**. Data are mean values standard deviation from three independent experiments.

Two-photon irradiation of M2PS and Au@M2PS was then conducted on MCF-7 breast cancer cells. The NPs were incubated at 40 μg.mL^−1^ for 20 h (the dose was chosen to induce significant cell death under irradiation and low effect without irradiation) with cancer cells in 384 multiwell glass bottomed plate. Then, the cells were irradiated or not with a confocal Carl Zeiss two-photon microscope (laser input power 3 W). The well was irradiated at the maximum power of the laser at 760 nm with the smallest objective (Carl Zeiss 10-fold magnification/objective 0.3 EC Plan-Neofluar). Three scans of 1.57 s were performed each in four different areas, without overlaps between irradiated areas. M2PS NPs caused 25% of selective cell-killing via TPE-PDT (Figure 4). Indeed, the diphenylbutadiene core of 2PS molecules act as an electron donor (Beaumont et al., 2008). However, Au@M2PS NPs produced 50% of selective cell death under irradiation. Given that the 2PS content are very similar in both NPs, such results strongly indicate that the TPE-PDT increase arose in the presence of AuNPs.

**Figure 5:**
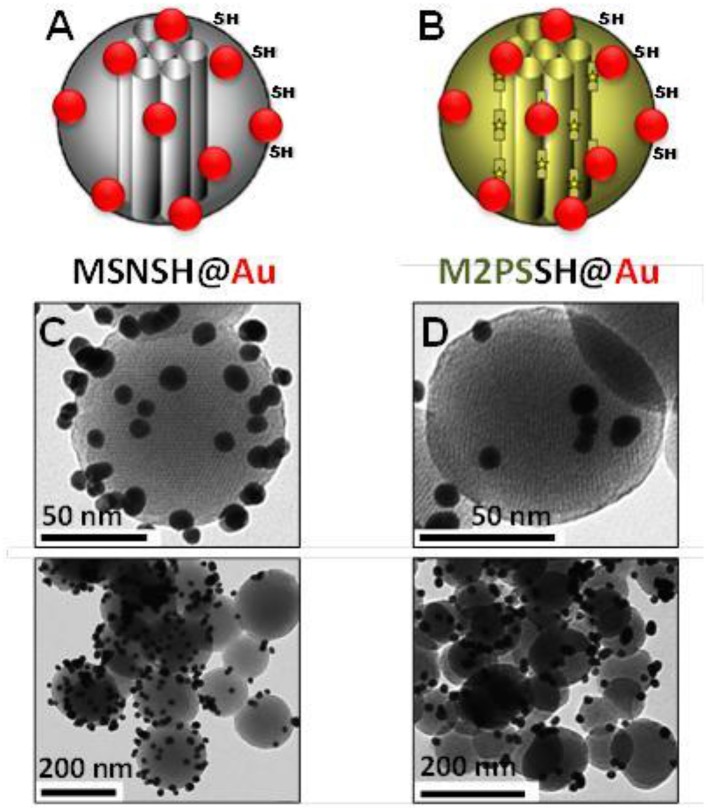
**Schematic representation (A,B) and TEM micrographs of MSNSH@Au (C), and M2PSSH@Au NPs (D)**. Yellow stars represent 2PS fragments.

**Figure 6:**
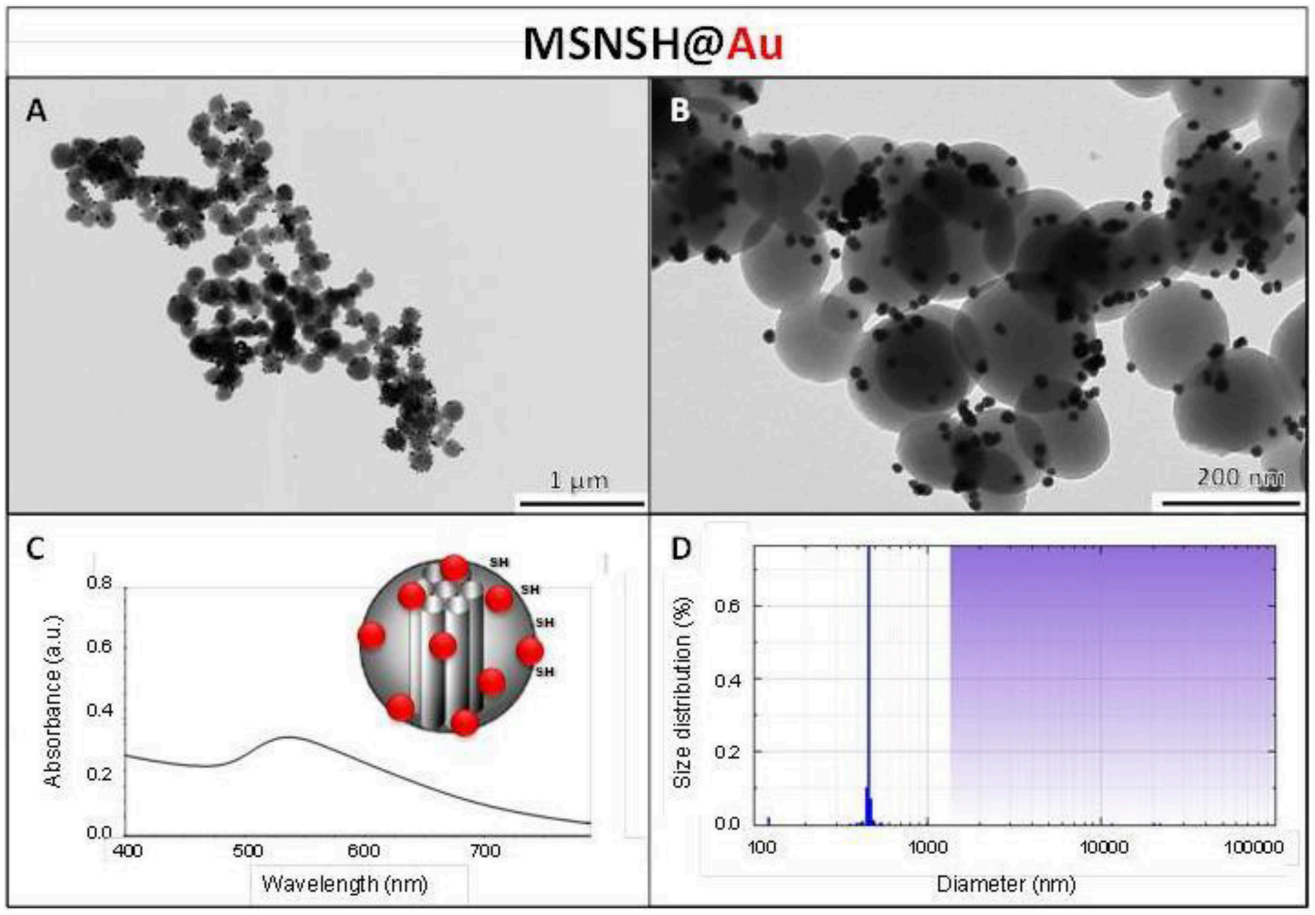
**MSNSH@Au NPs characterization via TEM images (A,B), uv-visible spectroscopy (C), and DLS size distribution (D)**.

**Figure 7:**
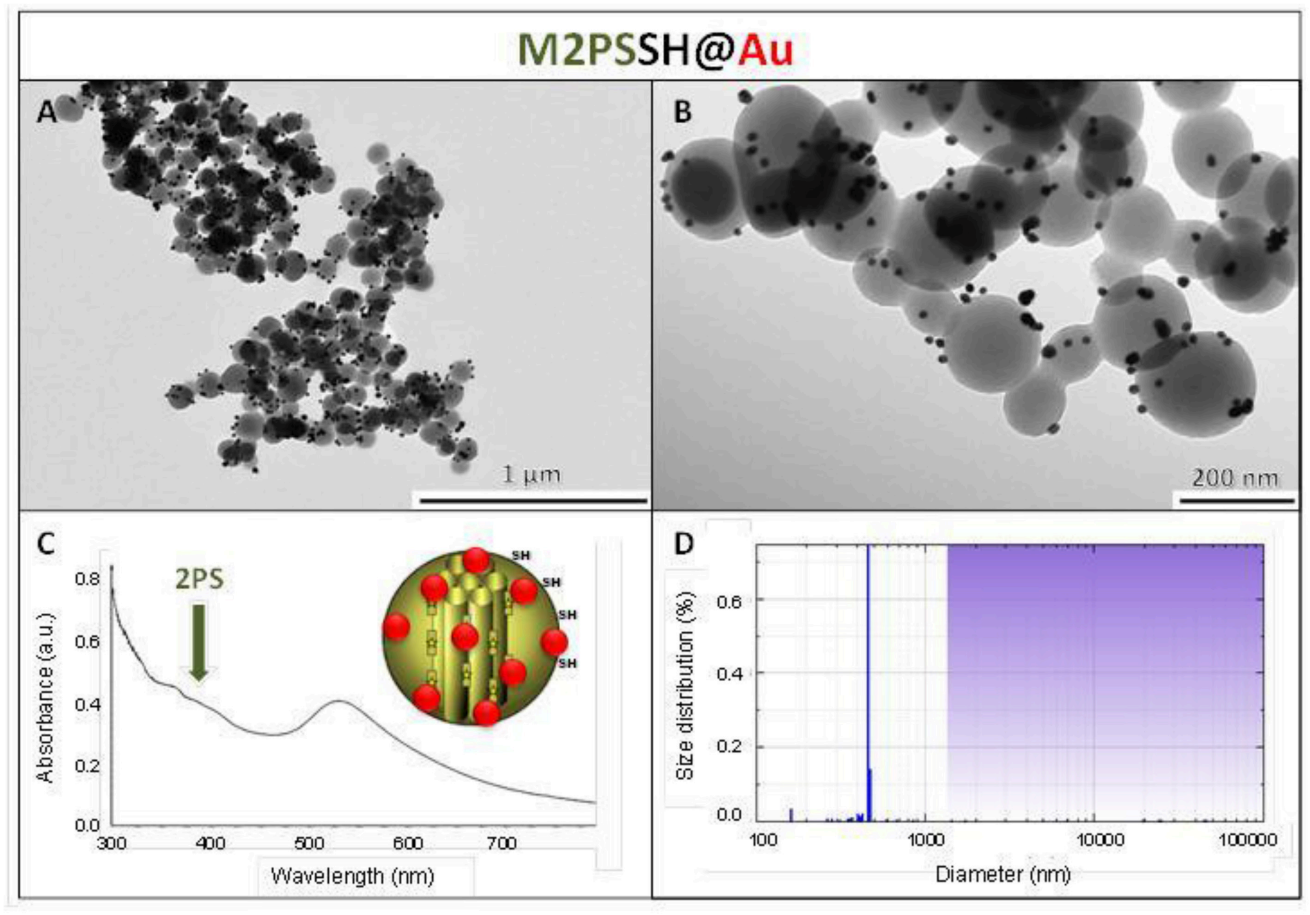
**M2PSSH@Au NPs characterization via TEM images (A,B), uv-visible spectroscopy (C), and DLS size distribution (D)**.

The influence of the surface functionalization of mesoporous NPs with AuNSs on TPE-imaging and PDT properties was then studied. Thiol groups, known for their excellent capability to chelate AuNPs, were incorporated in mesoporous particles in one-pot reactions affording MSNSH and M2PSSH NPs. Both reactions were performed with an initial addition of TEOS (and 2PS moieties in the case of M2PSSH), and after 6 min of condensation, mercaptopropyltrimethoxysilane (1:10 TEOS, v:v) was added to cover the growing outer surface of NPs. Surfactant-free MSNSH and M2PSSH NPs were fully characterized by TEM and DLS (Figures S1A–C, S2A–C), which showed nearly monodisperse porous 60–80 nm spherical particles. Uv-visible spectroscopy (Figure S2D) and elemental analysis confirmed the encapsulation of 8 wt% of 2PS fragments (Table S1). This procedure typically led to excellent surface areas in the order of 1200 m^2^.g^−1^ (Figures S1D, S2E). The presence of thiol groups was confirmed by the ν_C−H_ vibration modes at 2927 and 2856 cm^−1^ in MSNSH and M2PSSH, and the 2PS encapsulation was also validated by the ν_Si−C_ vibration at 1156 cm^−1^ and the aromatic ν_*C*−*H*_ modes at 3021 and 3062 cm^−1^ (Figure S3). Afterwards, thiol-functionalized NPs were mixed with AuNSs to elaborate surface decorated MSNSH@Au and M2PSSH@Au nanocarriers (see Figures 5A,B, 6, 7). The grafted AuNSs were readily visible on the surfaces of the porous MCM-41 nanomaterial by TEM images (see Figures 5C,D, 6A,B, 7A,B), and caused the appearance of the plasmonic band of gold nanocrystals at λ_max_ of 525 nm (see Figures 6C, 7C). Induced-coupled-plasma (ICP) measurements of the gold wt%, provided similar values of grafted nanocrystals (Au wt%_MSNSH@Au_ = 6.2, Au wt%_M2PSSH@Au_ = 5.4). Thus, the influence of AuNSs on gold-decorated mesoporous organosilica with or without 2PS could be investigated in cells.

**Figure 8:**
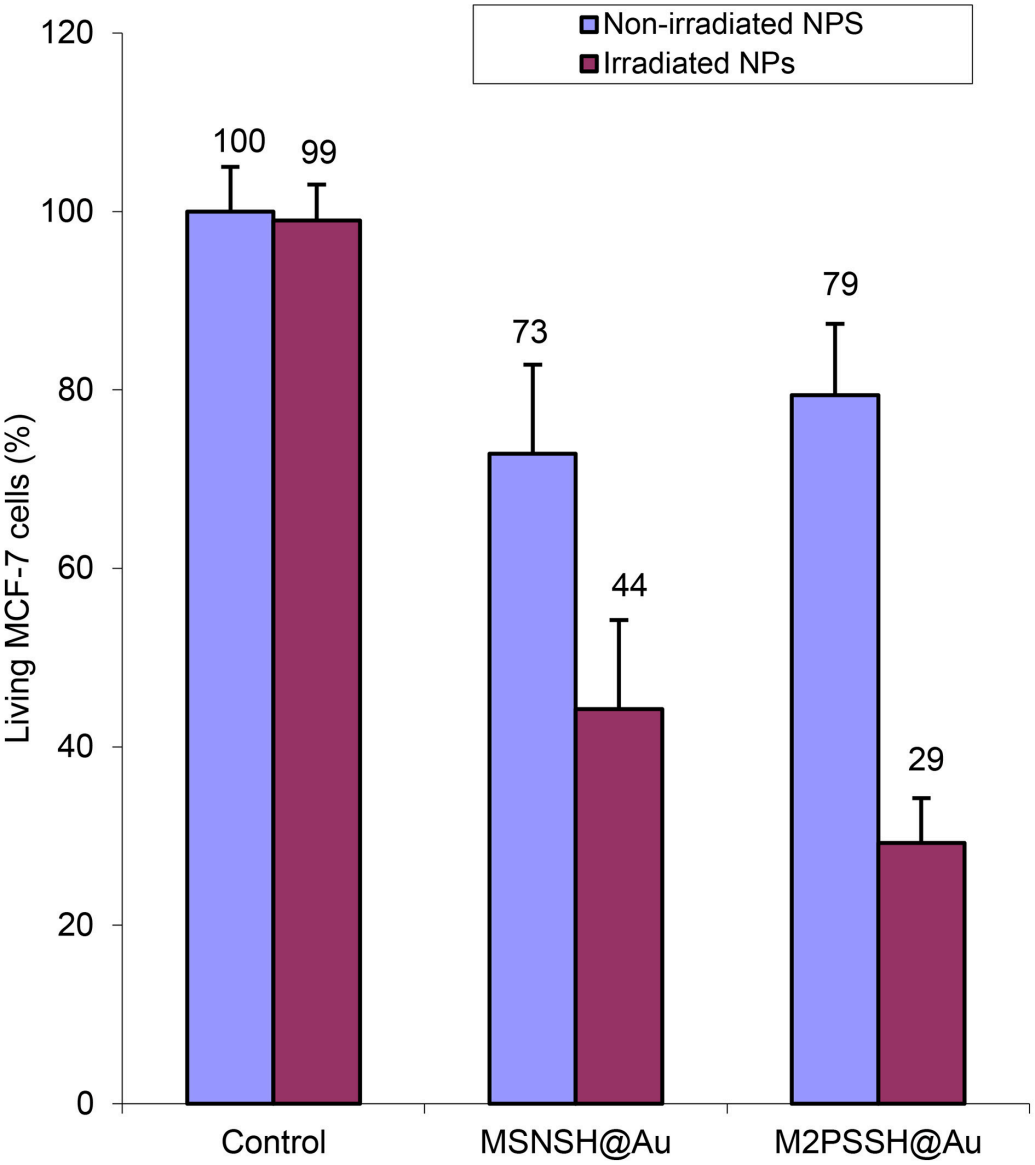
**TPE-PDT via MSNSH@Au and M2PSSH@Au NPs (40 μg.mL^−1^) in MCF-7 cells**. Data are mean values standard deviation of three different experiments.

Two-photon irradiations of MCF-7 incubated with MSNSH@Au and M2PSSH@Au NPs were then conducted in the same manner. Interestingly, 56% of selective cell killing was observed with MSNSH@Au under TPE (Figure 8). According to recent findings spatially close AuNSs behave like gold nanorods (AuNRs) (Jiang et al., 2013), and gold nanorods can produce singlet oxygen and can be used for TPE-PDT (Zhao et al., 2012, 2014; Chen et al., 2014). Furthermore, the irradiation of multifunctional M2PSSH@Au NPs caused 71% of cancer cell killing, thus proving the enhancement of TPE-PDT via a specific design of gold-mesoporous organosilica NPs. Note that, we also prepared AuNRs via a reported seed-growth method (ESI, see uv-visible spectra Figure S4), and such NPs were grafted on MSNSH and M2PSSH surfaces (Figures S5–S7). The aim was to assess the influence of the shape of AuNPs on the two-photon properties of materials, but unlike previous nanocomposites which displayed low or slight cytotoxicity at 40 mg.mL^−1^ (blue bars in Figures 4, 8), the resulting NPs were highly toxic though washed extensively (Figure S8), which is attributed to residual CTAB molecules from the synthetic process, as shown with the FTIR spectra of MSNSH@AuNRs (Figure S10). Taking advantage of the spatial proximity of AuNSs is thus particularly desirable in light of the potential toxicity of AuNRs.

**Figure 9:**
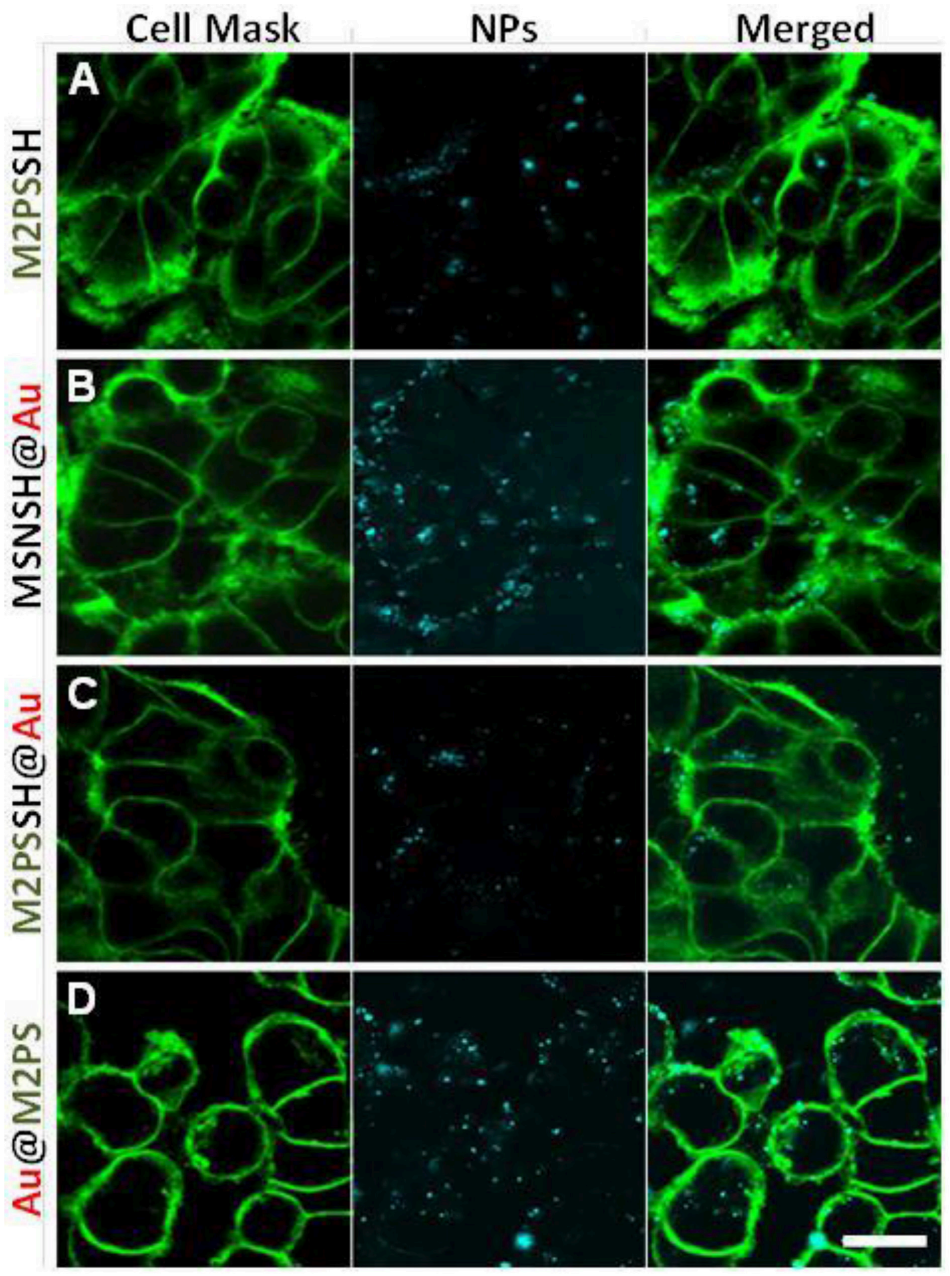
**TPE-fluorescence imaging of M2PS (A), MSNSH@Au (B), M2PSSH@Au (C), and Au@M2PS (D), NPs in MCF-7 cells**. Scale bar of 10 μm.

Two-photon imaging was finally studied with the various nanoplatforms (Figure 9). All the NPs were readily visible through TPE-fluorescence imaging which demonstrated the successful cellular uptake of NPs. Besides, it was found that MSNSH@Au also led to bright imaging (see Figure 9B), which further indicates the nanorods behavior of spatially close AuNSs, as occured in MSNSH@AuNRs and M2PSSH@AuNRs (Figure S9). The usefulness of gold nanospheres was further illustrated by the higher imaging capabilities of Au@M2PS and M2PSSH@Au NPs, when compared to M2PS NPs (see Figures 9B,D, and Figure 9A, respectively). Thus, the 2PS imaging properties can be combined with the scattering feature of spatially-close AuNSs to synergistically enhance the diagnostic function of M2PS NPs.

## Conclusions

In summary, a library of gold-mesoporous silica and organosilica has been prepared, fully characterized and systematically studied for two-photon imaging and therapy in cancer cells. M2PS and various AuNSs core-shells nanostructures were found to be biocompatible on MCF-7 breast cancer cell line. Moreover, the bright two-photon intracellular imaging of the NPs demonstrated the cellular uptake of all designed NPs. Experimental results demonstrated that spatially close AuNSs inside or on the surface of mesoporous NPs could be used to enhance TPE-PDT on MCF-7 cells with up to 71% of cell death. It is envisioned that such nanocomposites could be versatile theranostic nanovehicles thanks to the wide variety of cargos which could be loaded in their porous frameworks.

## Author contributions

JC made MSNs and gold systems, CQ made thiol functionalization, XC and MW performed click chemistry, OM and MB designed the electron donor and made the synthesis, MM performed two-photon imaging, MG and MGB performed TPE-PDT. LR analyzed the data and JOD wrote the paper with JC.

### Conflict of interest statement

The authors declare that the research was conducted in the absence of any commercial or financial relationships that could be construed as a potential conflict of interest.
