# Supplementary material for: Multifunctional Gold-Mesoporous Silica Nanocomposites for Enhanced Two-Photon Imaging and Therapy of Cancer Cells
Source: Front Mol Biosci. 2016 Feb 3;3:1. doi: 10.3389/fmolb.2016.00001 (PMC4737918; doi:10.3389/fmolb.2016.00001)
Supplement: Supplementary file 1 [file DataSheet1.docx]

SUPPLEMENTARY INFORMATION

Multifunctional Gold-Mesoporous Silica Nanocomposites for Enhanced Two-Photon Imaging and Therapy of Cancer Cells

Jonas G. Croissant,^1^* Christian Qi,^1^ Marie Maynadier,^2^ Xavier Cattoёn,^3^ Michel Wong Chi Man,^1^ Laurence Raehm,^1^ Olivier Mongin,^4^ Mireille Blanchard-Desce,^5^ Marcel Garcia,^6^ Magali Gary-Bobo ^6^* and Jean-Olivier Durand^1^*

^1^Institut Charles Gerhardt Montpellier, UMR-5253 CNRS-UM2-ENSCM-UM1, case courrier 1701 Place Eugène Bataillon, F-34095, Montpellier Cedex 05, France [durand@um2.fr](mailto:durand@um2.fr), jonasc@chem.ucla.edu
^2^ NanoMedSyn, 2 - Faculté de Pharmacie, 15, Avenue Charles Flahault, 34093 Montpellier Cedex 05, France

^3^Institut NEEL, CNRS, and Université Grenoble Alpes, F-38042 Grenoble, France.

^4^Institut Des Sciences Chimiques de Rennes, CNRS UMR 6226 Université Rennes 1 Campus Beaulieu F-35042 Rennes Cedex, (France)

^5^Univ. Bordeaux, Institut des Sciences Moléculaires, UMR CNRS 5255, 351 Cours de la Libération, F-33405 Talence Cedex, (France)

^6^Institut des Biomolécules Max Mousseron UMR 5247 CNRS; UM 1; UM 2 - Faculté de Pharmacie, Université Montpellier, 15, Avenue Charles Flahault, 34093 Montpellier Cedex 05, France. magali.gary-bobo@inserm.fr


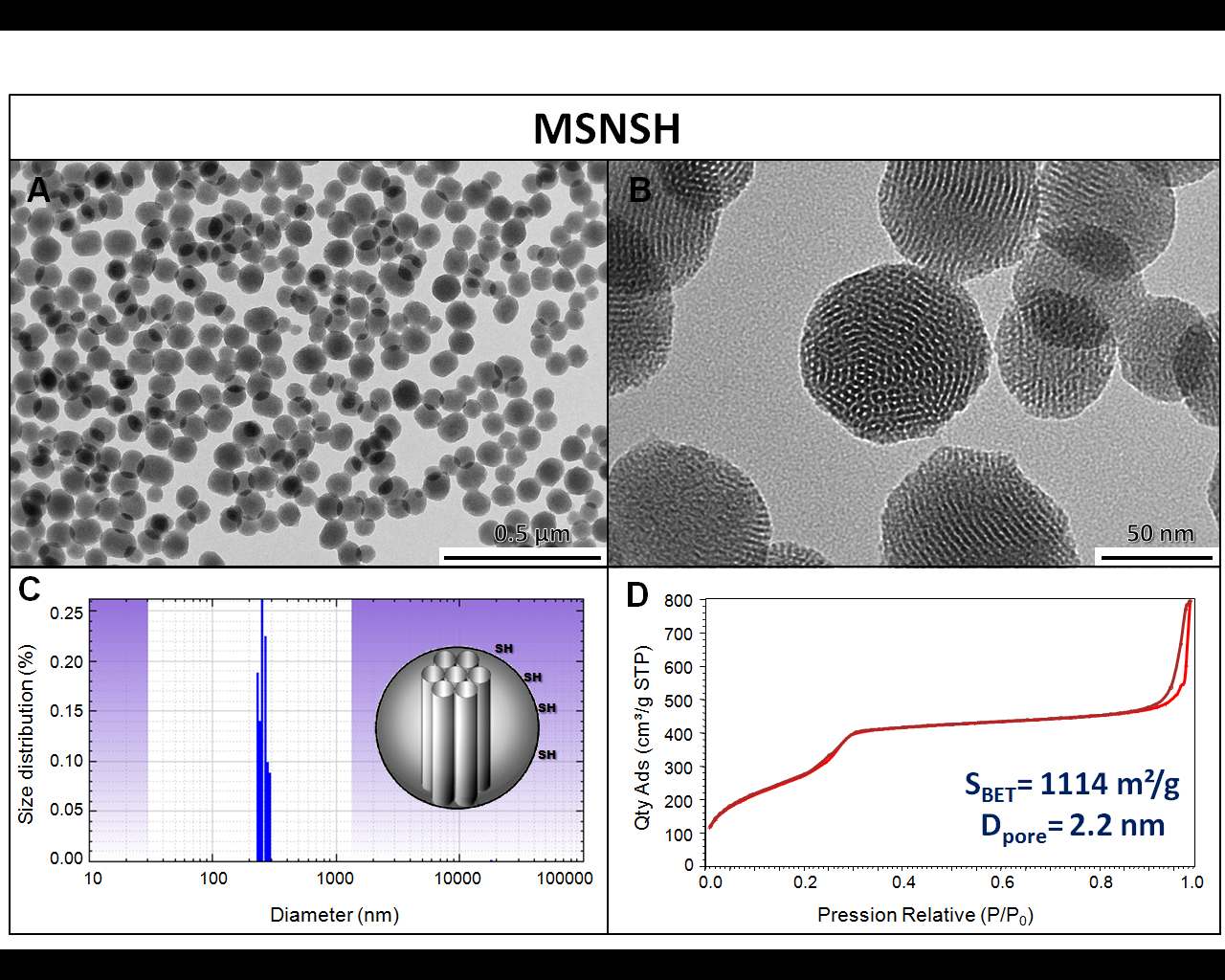


Figure S1. MSNSH NPs characterization via TEM images (A-B), N_2_-adsorption-desorption (C), and DLS size distribution (D).


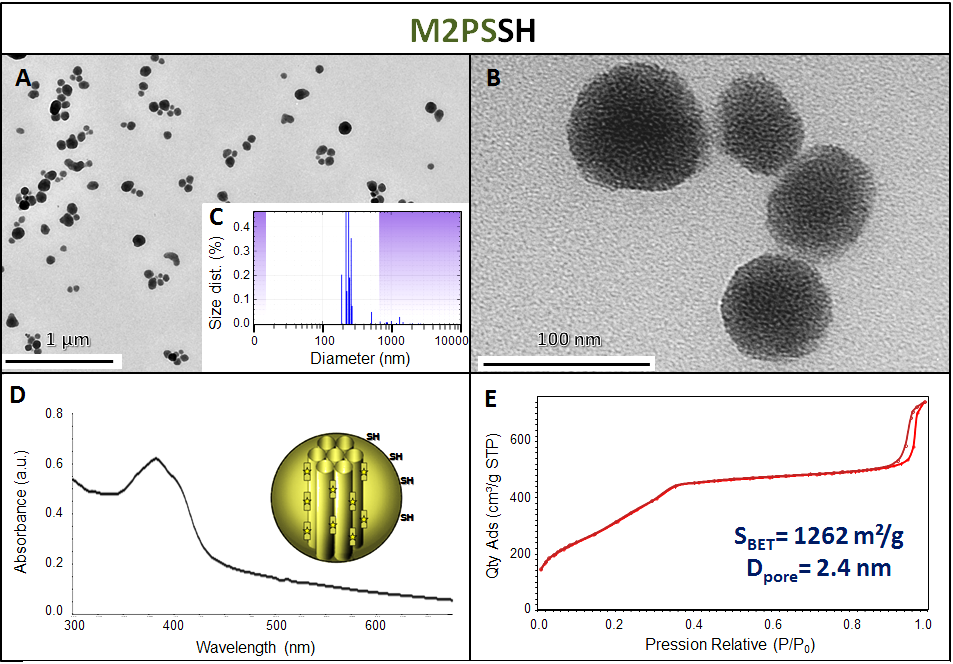


Figure S2. M2PSSH NPs characterization via TEM images (A-B), DLS size distribution (C), N_2_-adsorption-desorption (D), and uv-visible spectroscopy (E).

**Table S1***.* 2PS weight percent determination in the NPs.

| Sample | **NPs N wt% ^[a]^** | **NPs 2PS wt% ^[b]^** |
| --- | --- | --- |
| **M2PS** | 3.5 | 16.7 |
| **Au@M2PS** | 4.3 | 20.5 |
| **M2PSSH@Au** | 1.8 | 8.6 |

[a] Elemental analysis by combustion measurements of the NPs. [b] Determination based on the nitrogen wt% in the 2PS molecules.

Table S2. Gold weight percent determination in NPs.

| Sample | **AuNSs wt%** |
| --- | --- |
| **Au@M2PS** | 4.7 **^[a]^** |
| **MSNSH@Au** | 6.2 **^[b]^** |
| **M2PSSH@Au** | 5.4 **^[b]^** |

[a] Energy dispersive spectrometry analysis.

[b] Inductively coupled plasma atomic emission spectroscopy.


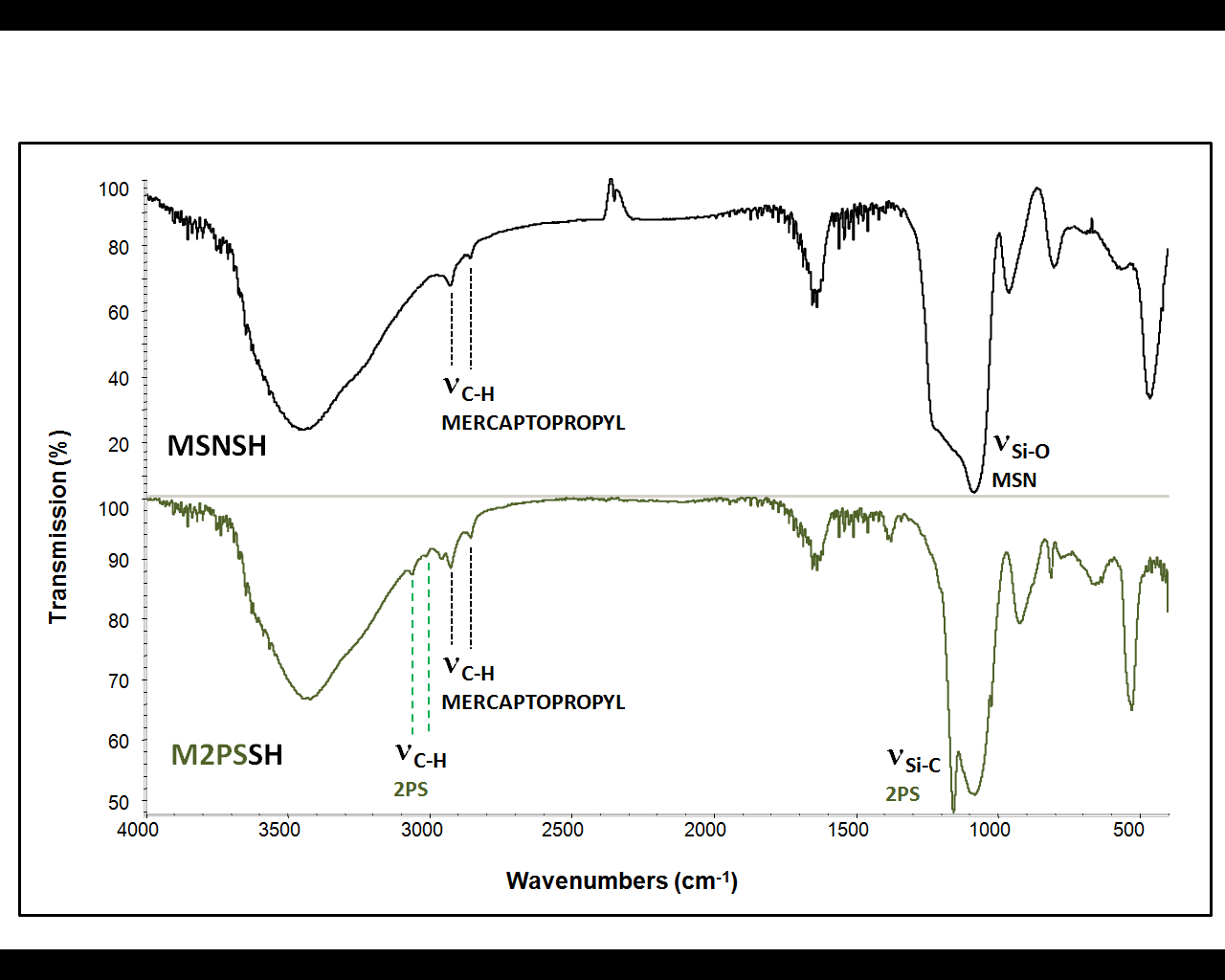


Figure S3. FTIR spectra of MSNSH and M2PSSH NPs, confirming the thiol co-condensation and the 2PS encapsulation.


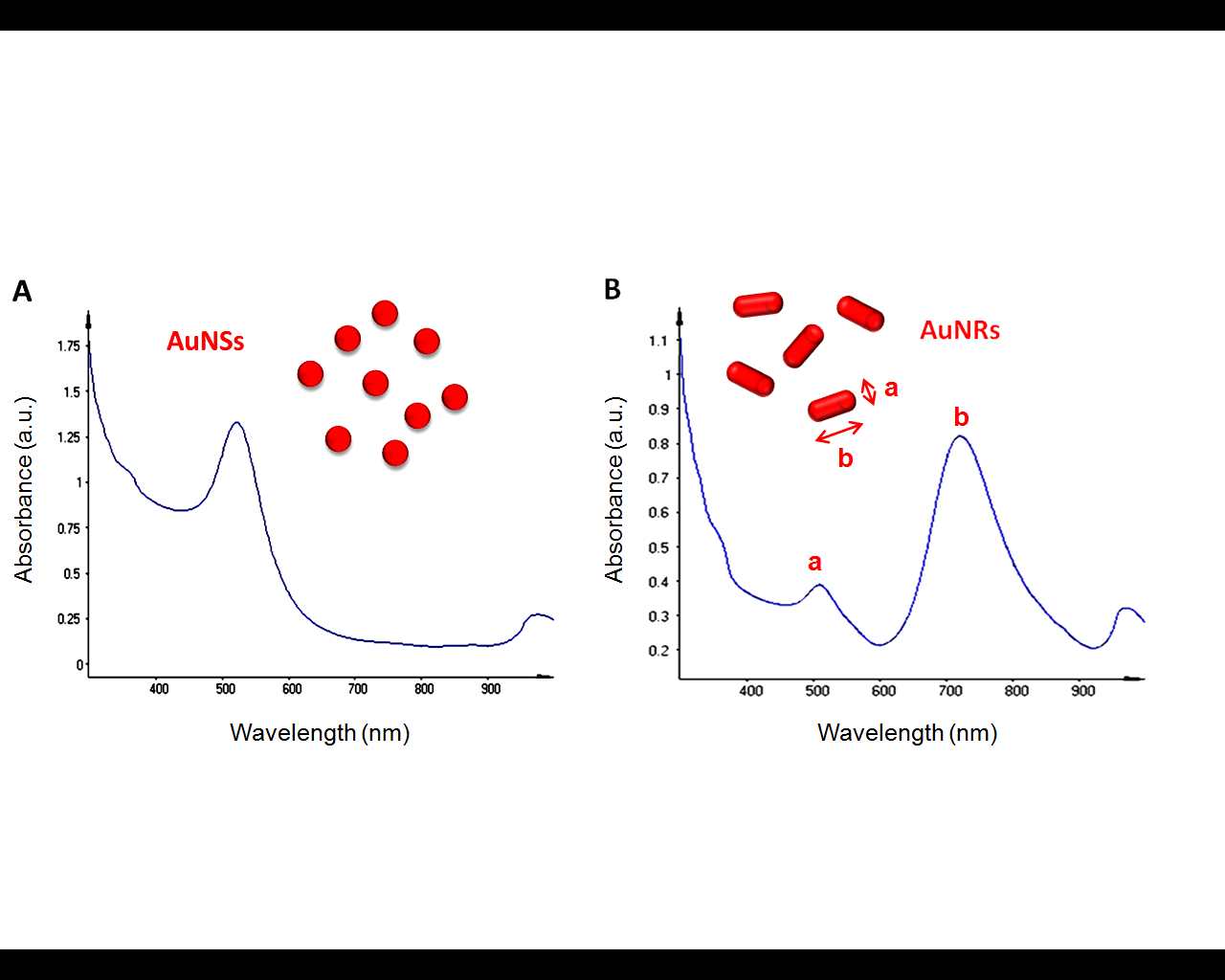


Figure S4. Uv-visible spectra of gold nanospheres (A), and gold nanorods (B).


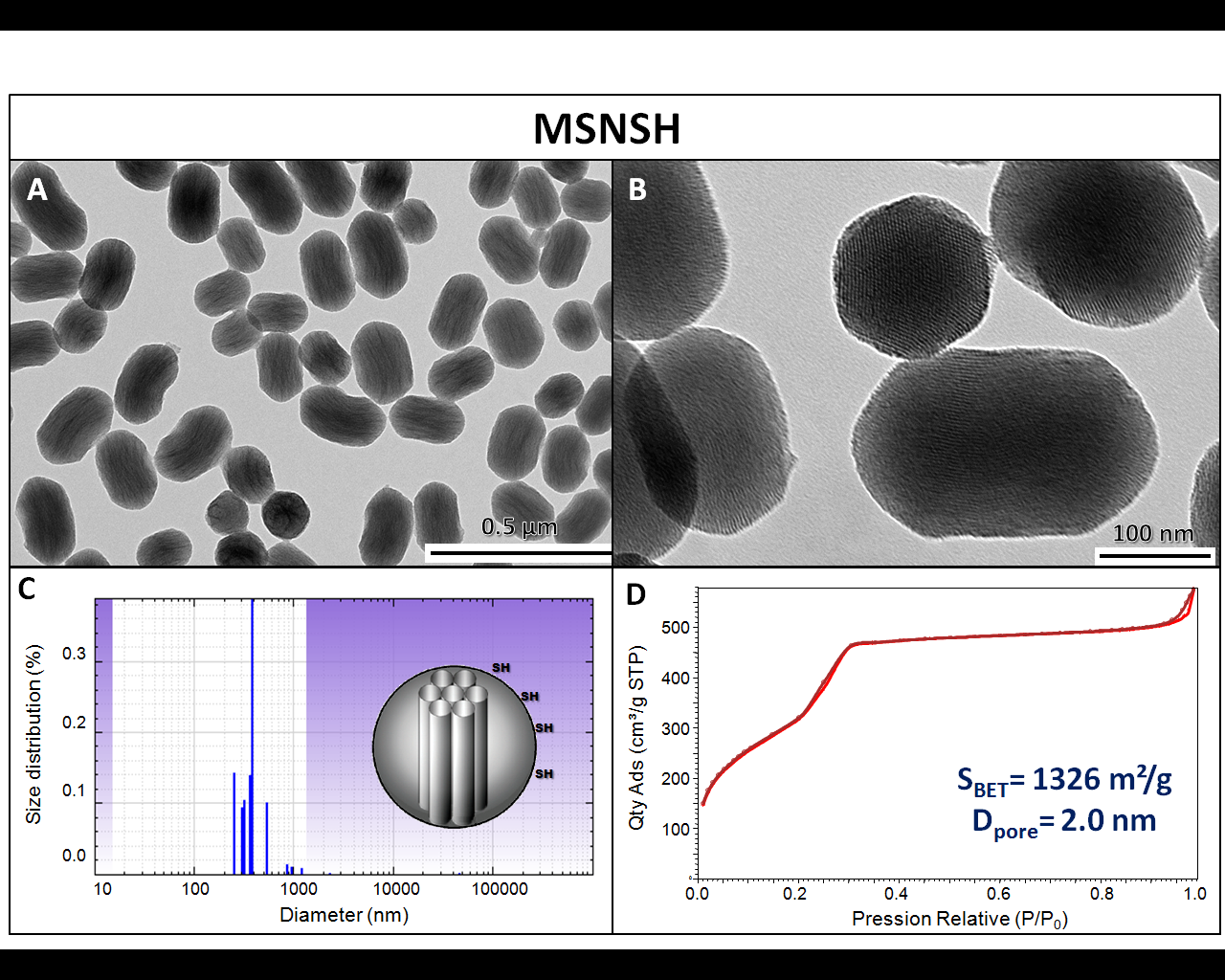


Figure S5. MSNSH NPs characterization via TEM (A-B), DLS (C), and N_2_-adsorption-desorption technique (D).


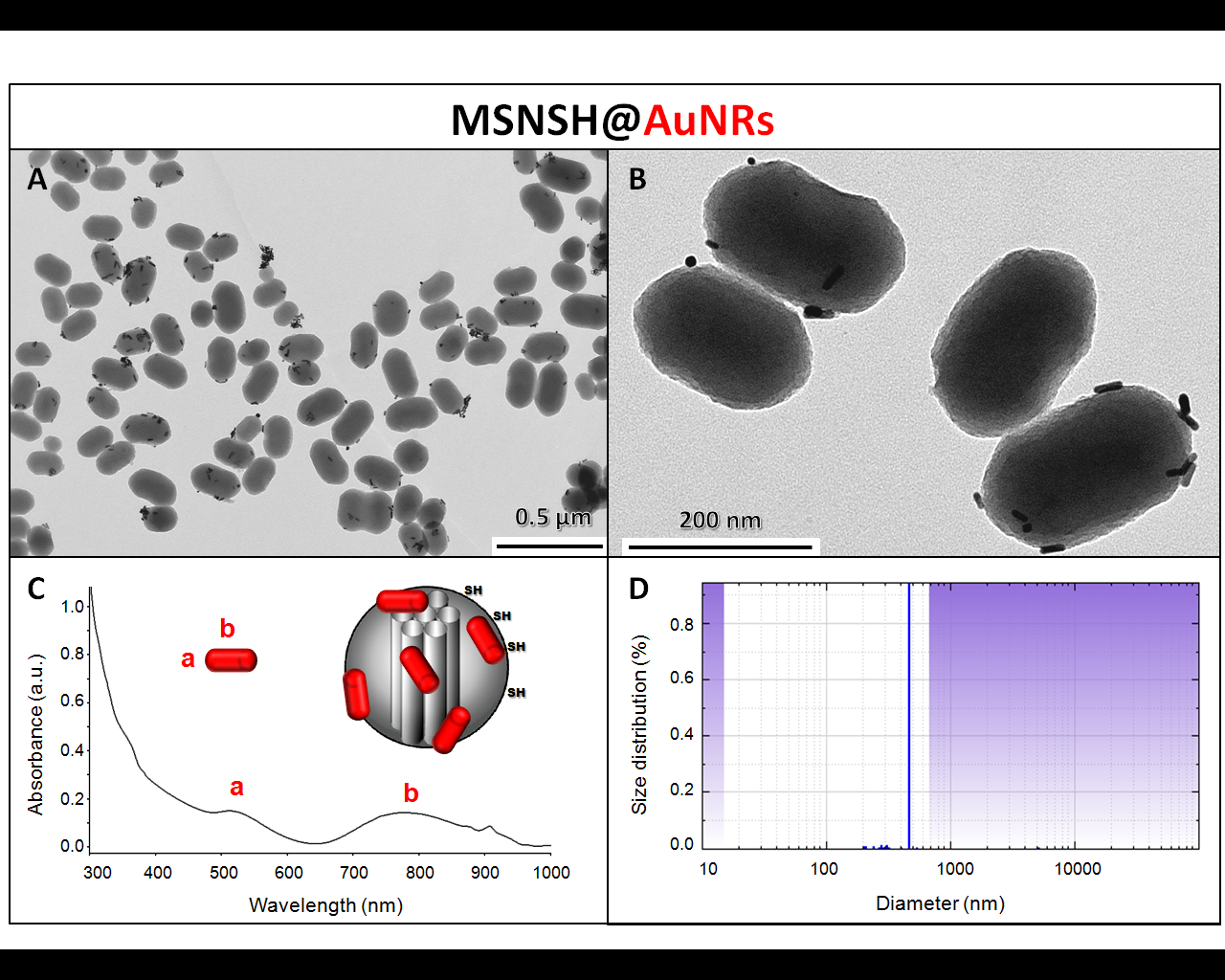


Figure S6. MSNSH@AuNRs NPs characterization via TEM (A-B), uv-visible spectroscopy (C), and DLS (D).


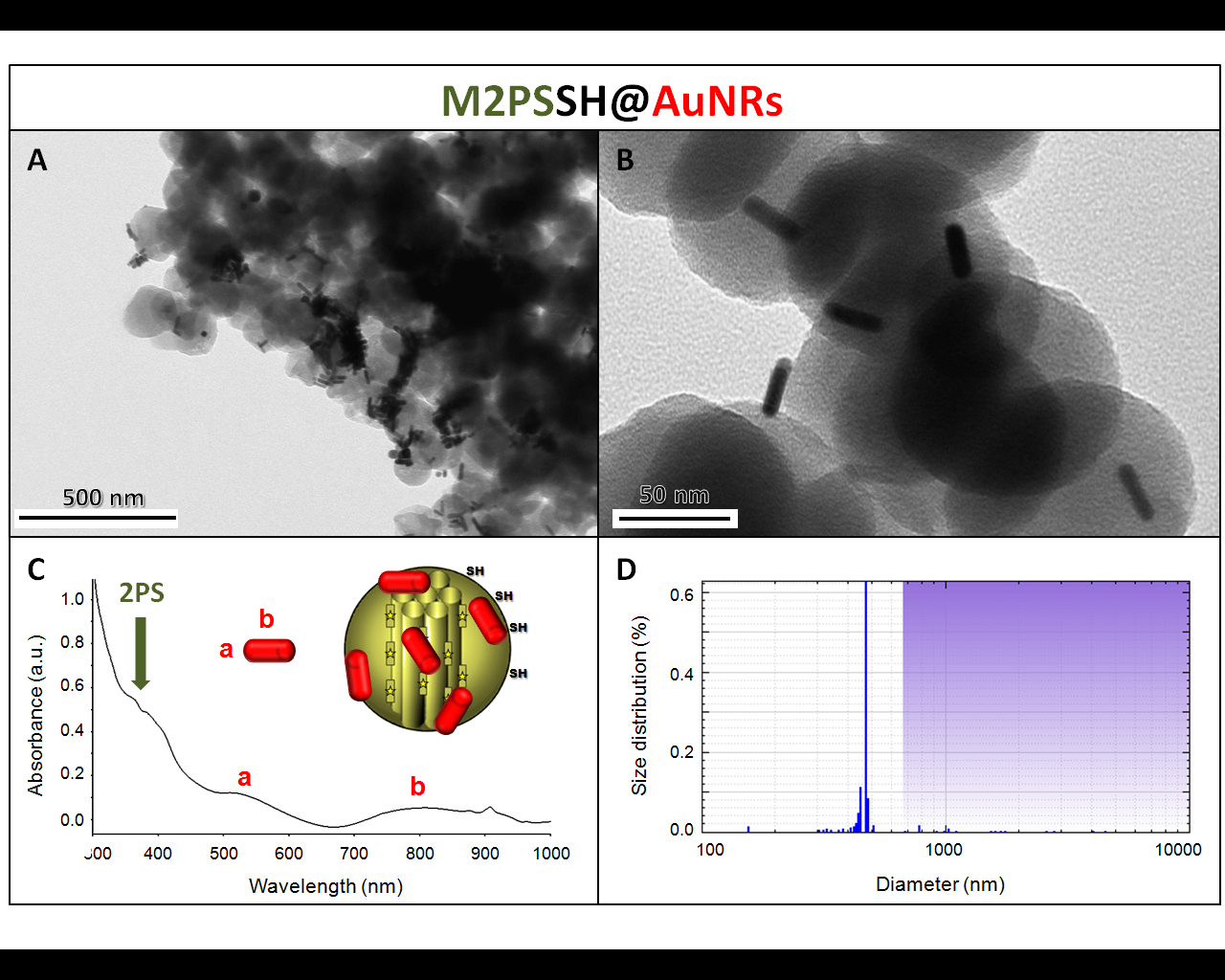


Figure S7. M2PSSH@AuNRs NPs characterization via TEM (A-B), uv-visible spectroscopy (C), and DLS (D).


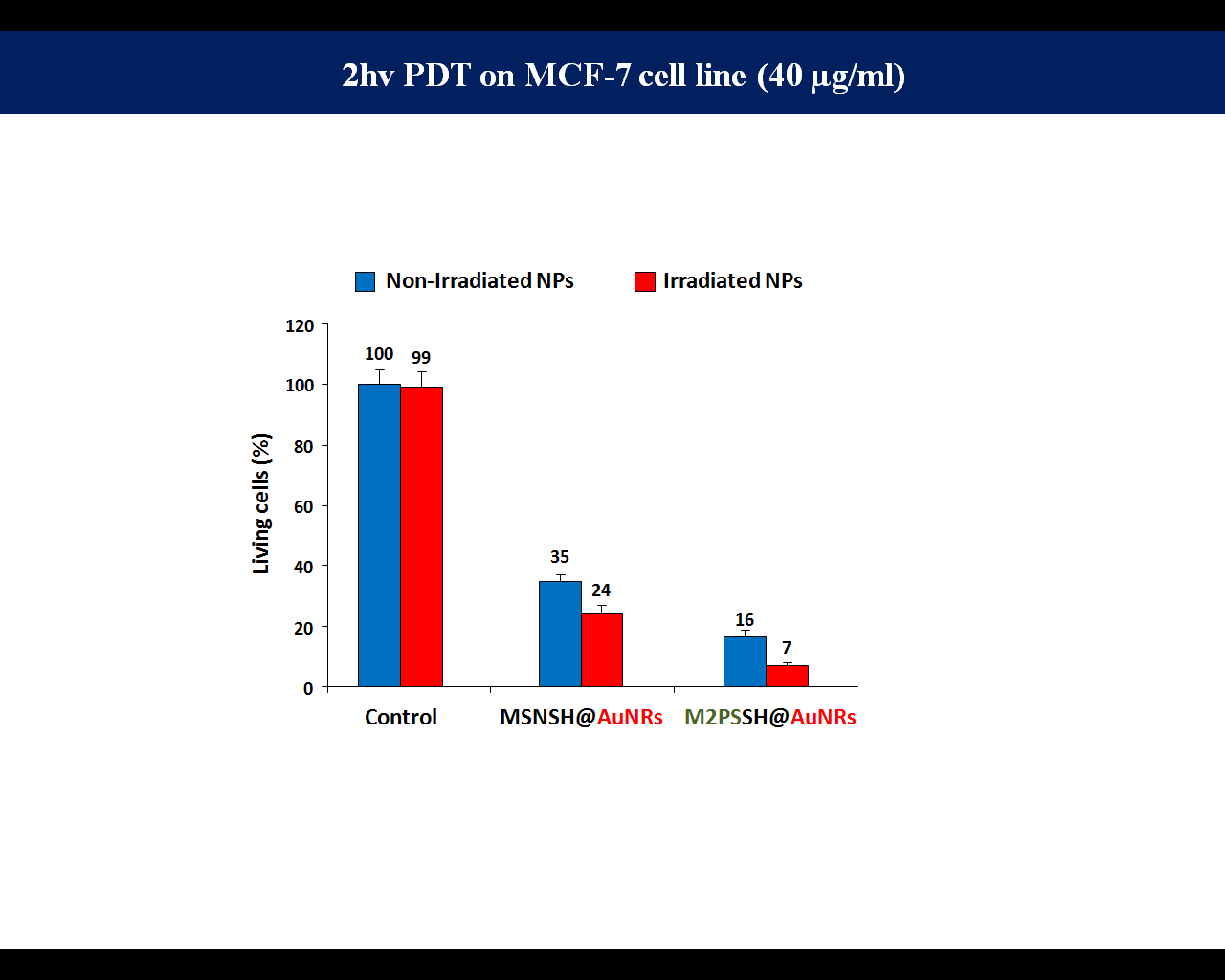


**Figure S8**. Cell viability study of two-photon irradiated MSNSH@AuNRs and M2PSSH@AuNRs in MCF-7 breast cancer cells at 40 µg.mL^-1^. Data are mean values standard deviation from three independent experiments.


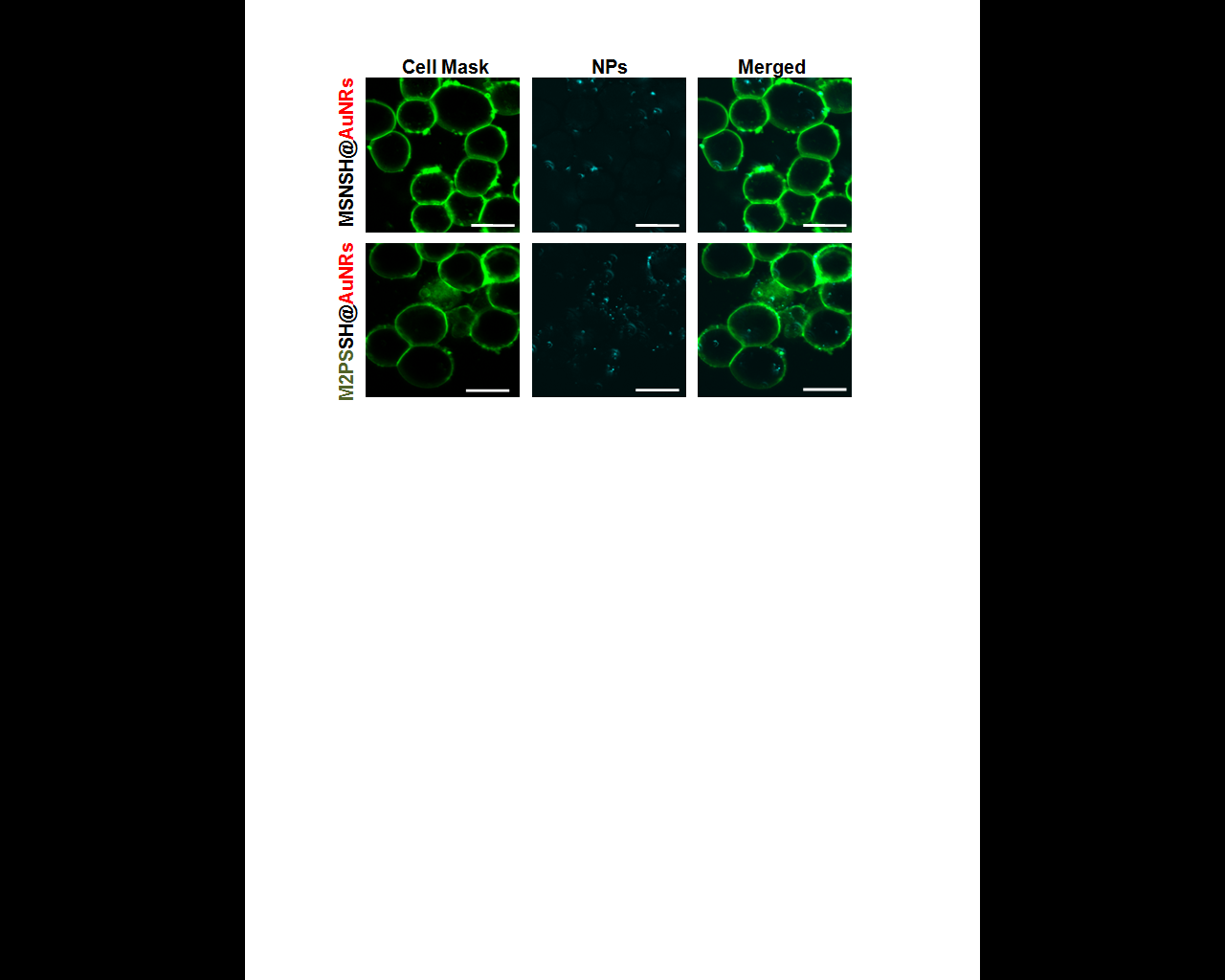


**Figure S9**. Two-photon fluorescence imaging of MSNSH@AuNRs and M2PSSH@AuNRs in MCF-7 breast cancer cells at 40 µg.mL^-1^ (Scale bar 10 µm).


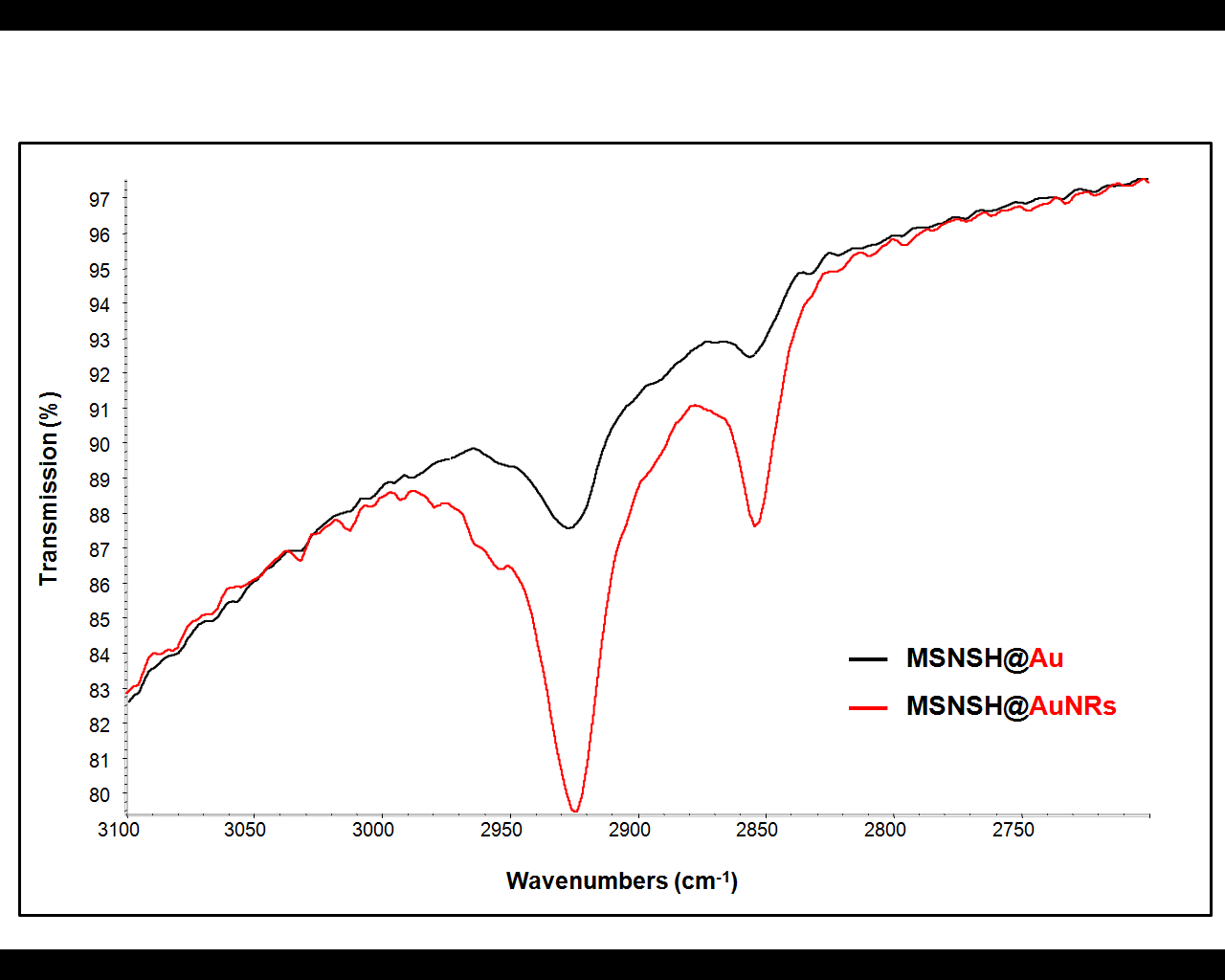


Figure S10. FTIR spectra of MSNSH@Au and MSNSH@AuNRs, validating the CTAB cytotoxicity origin postulated in MSNSH@AuNRs. The higher intensity of the C-H stretching modes in MSNSH@AuNRs could not be attributed to mercaptopropyl groups (which are also present in MSNSH@Au), and are most likely arising from residual CTAB molecules used in the AuNRs synthesis.
